# Direct Clipping for Active Colonic Diverticular Bleeding Using an Endoclip with Re-grasping Function

**DOI:** 10.5152/tjg.2024.24004

**Published:** 2024-09-01

**Authors:** Takaaki Kishino, Maiko Yamakawa

**Affiliations:** Department of Gastroenterology and Hepatology, Center for Digestive and Liver Diseases, Nara City Hospital, Nara, Japan

## CASE PRESENTATION

Colonic diverticular bleeding (CDB) is the most common cause of lower gastrointestinal bleeding. Management of CDB is clinically important because severe cases require transarterial embolization (TAE) or surgical intervention.^1^ Endoscopic hemostasis for CDB with stigmata of recent hemorrhage potentially prevents rebleeding.^2^ Among the various endoscopic treatments for CDB, clipping is widely used worldwide because of its simplicity and theoretical advantage of causing less damage to adjacent tissues.^1,3^ Clipping methods for CDB are classified as direct or indirect, and direct clipping has demonstrated significantly lower rates of rebleeding compared with indirect clipping.^4^ However, direct clipping for active CDB is challenging and less effective, because the bleeding point is obscured by bleeding.^4^ Recently, a novel endoclip device with re-grasping function has been developed. We report a case in which active CDB was successfully treated by direct clipping using this clip. Written informed consent was obtained from the patient who agreed to take part in the study.

**Figure 1.**
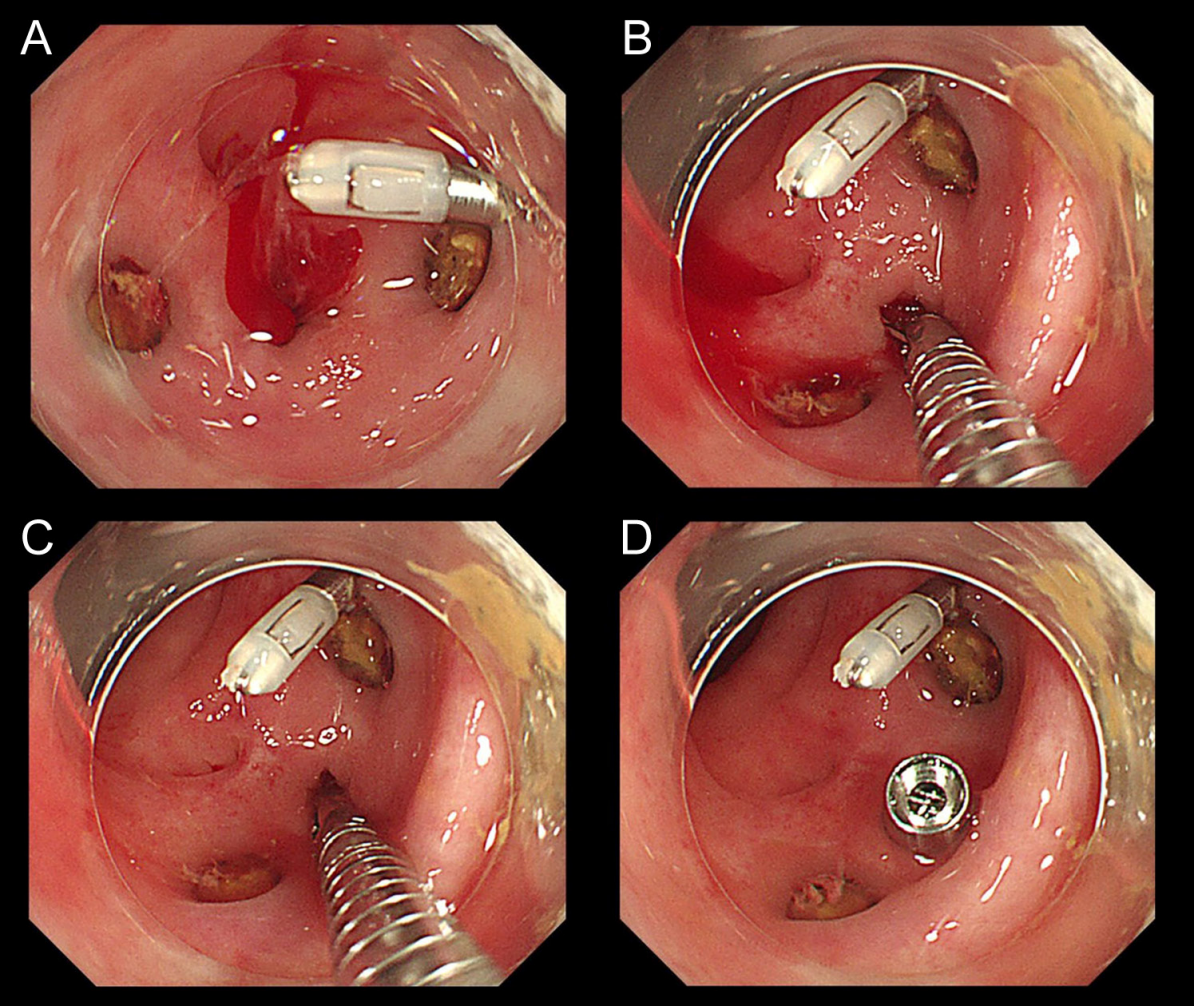
(A) Identifying a diverticulum with active bleeding in the sigmoid colon. (B) Inserting the clipping device into the diverticulum and opening it. (C) Grasping the base of the diverticulum. (D) Releasing the clip after confirming hemostasis. Bleeding did not recur after treatment.

An 82-year-old woman who was taking edoxaban for paroxysmal atrial fibrillation presented with hematochezia. After admission, an urgent colonoscopy was performed. We identified a diverticulum with active bleeding in the sigmoid colon (Figure 1). We selected direct clipping for endoscopic hemostasis because endoscopic band ligation (EBL) was considered challenging due to the presence of multiple diverticula (Figure 1). We performed direct clipping using an endoclip device with re-grasping function according to the above procedure, and the bleeding was successfully stopped. Bleeding did not recur after the treatment.

### TECHNIQUE

**Figure 2.**
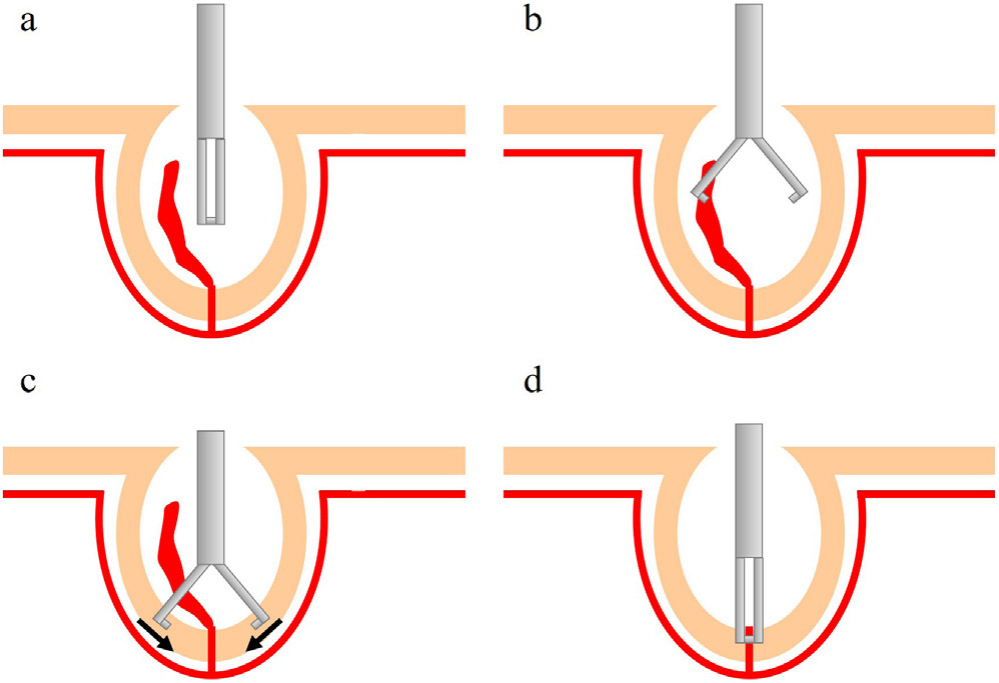
Schema of direct clipping using an endoclip with re-grasping function. (A) Inserting the clipping device into the diverticulum. (B) Opening the clipping device. (C) Capturing the base of the diverticulum. (D) Releasing the clip after confirming that hemostasis is achieved. If bleeding continues, the endoscopist opens the clip and changes the point of grasping.

The procedure of endoscopic hemostasis for CDB using the re-grasping function is as follows. First, an endoclip device is inserted into the diverticulum. Next, the endoscopist opens the clip in the diverticulum and captures the base of the diverticulum. Finally, the clip is released after confirming that hemostasis has been achieved (Figures 1 and 2, Video*).

## CONCLUSION

When endoscopic clipping for CDB fails, EBL, TAE, or surgical intervention is selected as an alternative treatment, but these treatments are more invasive than endoscopic clipping. Because conventional endoscopic clipping for active CDB is challenging, an endoclip device with re-grasping function is considered useful for direct clipping. This clip has two key advantages. Firstly, the clip can be opened inside the diverticulum and can grasp the base of the diverticulum, even if the diverticular orifice is small. Secondly, grasping can be repeated until hemostasis is confirmed, even if the bleeding point is not visible, for example, due to active bleeding. If the bleeding is not stopped with this method, EBL can be additionally performed (the EBL-C method).^5^ In conclusion, direct clipping is acceptable even for active CDB if an endoclip with re-grasping function is used.

*The video file linked to this article is available in the online version of the journal. Or you can utilize the QR code provided on this page to gain access.
